# *In silico* Analysis of SARS-CoV-2 ORF8-Binding Proteins Reveals the Involvement of ORF8 in Acquired-Immune and Innate-Immune Systems

**DOI:** 10.3389/fmed.2022.824622

**Published:** 2022-02-01

**Authors:** Hisashi Takatsuka, Muhamad Fahmi, Kotono Hamanishi, Takuya Sakuratani, Yukihiko Kubota, Masahiro Ito

**Affiliations:** ^1^Department of Bioinformatics, College of Life Sciences, Ritsumeikan University, Kusatsu, Japan; ^2^Research Department, Research Institute for Humanity and Nature, Kyoto, Japan; ^3^Research Organization of Science and Technology, Ritsumeikan University, Kusatsu, Japan

**Keywords:** SARS-CoV-2, phylogenetic profiling, immune evasion, COVID-19 pathogenesis, ORF8 accessory gene

## Abstract

SARS-CoV-2 is the causative agent of a new type of coronavirus infection, COVID-19, which has rapidly spread worldwide. The overall genome sequence homology between SARS-CoV-2 and SARS-CoV is 79%. However, the homology of the ORF8 protein between these two coronaviruses is low, at ~26%. Previously, it has been suggested that infection by the ORF8-deleted variant of SARS-CoV-2 results in less severe symptoms than in the case of wild-type SARS-CoV-2. Although we found that ORF8 is involved in the proteasome autoimmunity system, the precise role of ORF8 in infection and pathology has not been fully clarified. In this study, we determined a new network of ORF8-interacting proteins by performing *in silico* analysis of the binding proteins against the previously described 47 ORF8-binding proteins. We used as a dataset 431 human protein candidates from Uniprot that physically interacted with 47 ORF8-binding proteins, as identified using STRING. Homology and phylogenetic profile analyses of the protein dataset were performed on 446 eukaryotic species whose genome sequences were available in KEGG OC. Based on the phylogenetic profile results, clustering analysis was performed using Ward's method. Our phylogenetic profiling showed that the interactors of the ORF8-interacting proteins were clustered into three classes that were conserved across chordates (Class 1: 152 proteins), metazoans (Class 2: 163 proteins), and eukaryotes (Class 3: 114 proteins). Following the KEGG pathway analysis, classification of cellular localization, tissue-specific expression analysis, and a literature study on each class of the phylogenetic profiling cluster tree, we predicted that the following: protein members in Class 1 could contribute to COVID-19 pathogenesis via complement and coagulation cascades and could promote sarcoidosis; the members of Class 1 and 2, together, may contribute to the downregulation of Interferon-β; and Class 3 proteins are associated with endoplasmic reticulum stress and the degradation of human leukocyte antigen.

## Introduction

The first case of COVID-19 caused by SARS-CoV-2 was reported in late December 2019 in Wuhan, China (1). The virus quickly spread, resulting in a devastating worldwide pandemic that, 2 years later, continues to be a global crisis. Accordingly, a more complete understanding of the pathology of COVID-19 is a top research goal. It is known that SARS-CoV-2 encodes several structural and accessory proteins that support the proliferation and infective properties of the virus. In 2020, we reported that the SARS-CoV-2 accessory gene open reading frame 8 (ORF8) is a SARS-CoV-2-specific gene with low homology between SARS-CoV-2 and SARS-CoV (2).

ORF8 is encoded in the most variable region of the SARS-CoV-2 genome. Therefore, the open reading frame of ORF8 has been identified as a hotspot region for mutations and deletions during the early onset of human-to-human transmission (2–4). In the early stages of the pandemic, a mutant SARS-CoV-2 without the ORF8 protein was identified, evidenced by the deletion of 382 nucleotides (Δ382) in this hotspot region (5, 6). The patients that were infected with this ORF8 deletion mutant did not develop hypoxia (requiring oxygen supplementation), which is a typical symptom of COVID-19, and their clinical symptoms were milder than those of SARS-CoV-2 cases caused by the wild-type ORF8 (6). The SARS-CoV-2 delta variant, with an 872 deletion within the ORF8 coding region, also spread worldwide. There has been a higher hospital admission or emergency care risk for patients infected with the COVID-19 delta variant than for those infected with the alpha variant (7). Thus, understanding the ORF8 function is important for both clinical and anti-outbreak purposes. One target protein that affects the symptoms of ORF8-defective SARS-CoV-2-infected patients is the human leukocyte antigen (HLA) class I protein, which plays a significant role in the immune system and is a proteolytic target in ORF8-dependent mechanisms. Consequently, SARS-CoV-2-infected cells do not properly activate cytotoxic T cells, resulting in insufficient virus elimination (8).

Although the ORF8 protein is likely to be involved in the progression of COVID-19 pathology, the precise function of ORF8 and its pathogenic mechanism remain elusive. Previously, we explored the function of ORF8 using bioinformatics and found that it is localized in the endoplasmic reticulum and the secretory/intercellular compartments, which affect the endoplasmic reticulum and immunity systems, respectively (9). It has been shown in laboratory experiments that SARS-CoV-2 ORF8 induces endoplasmic reticulum stress by causing the divergence of activating transcription factor 6 (ATF6) and inositol-requiring enzyme 1 (IRE1) in the endoplasmic reticulum stress pathway (10). Furthermore, it has been shown that ORF8 promotes the expression of inflammatory factors by activating the IL-17 signaling pathway associated with immunity and acts as a causal factor of the cytokine storm in COVID-19 pathology (11).

We performed, in the present study, an *in silico* analysis of 431 proteins that bind to 47 ORF8-binding proteins to determine a new network of proteins involved in ORF8-dependent COVID-19 pathology. Our phylogenetic profiling showed that the interactors of ORF8-interacting proteins were clustered into three classes that are conserved across chordates (Class 1: 152 proteins), metazoans (Class 2: 163 proteins), and eukaryotes (Class 3: 114 proteins). Following the KEGG pathway analysis, the classification of cellular localization, tissue-specific expression analysis, and literature studies on each class of the phylogenetic profiling cluster tree, we present insight into the mechanism by which ORF8 contributes to COVID-19 pathogenesis and SARS-CoV-2 immune evasion.

## Materials and Methods

### Construction of Datasets

The information on ORF8 interacting human proteins was retrieved from a report in which 47 proteins were shown to bind with SARS-CoV-2 ORF8, as evidenced by affinity purification and mass spectrometry in HEK293T cells (12). The physical interaction partners of these proteins were then identified using the STRING database (Retrieved 2021_07, version11.5) (13). The amino acid sequence, protein function, cellular localization, and KEGG-ID information were added to the dataset from UniProt KB/Swiss-Prot, which was manually annotated in UniProt (release 2021_03) (14).

### Phylogenetic Profile Analysis

Phylogenetic profile analyses of the 47 human ORF8-interacting proteins and their interactors were performed on 587 genome-decoded species registered in the KEGG database (Retrieved 2021_07) (15). The KEGG Ortholog Cluster was used to determine the orthologs of the target proteins in each species (16). The KEGG Ortholog Cluster is a tool that aligns each amino acid sequence, using the Similarity Waterman algorithm, and classifies it as an ortholog when the score fulfills species criteria (score ≥ 150 and symmetric similarity measures). The generated phylogenetic profile was calculated using Euclidean distance with or without an ortholog of protein distance, and hierarchical clustering was performed using Ward's method. The clusters were characterized for tissue-specific gene expression patterns and intracellular localization in tissues and cells. Phylogenetic profile analysis, which uses a bit pattern that indicates the presence or absence of target protein orthologs in other species, is a method for predicting protein functions, interactions, and co-evolution from the perspective of phylogenetic evolution.

### Gene Ontology Enrichment Analysis

To identify the potential biological function differences for each of the phylogenetically clustered classes, the R package “clusterProfiller” (version3.16.1) was used to perform gene ontology (GO) analysis using the following thresholds: a *p*-value cut-off of 0.01 and a *q*-value cut-off of 0.05. Enrichment analysis was performed for each phylogenetic class (17).

### Classification of Cellular Localization

Cellular localization was classified based on GO annotation retrieved from UniProt KB/Swiss-prot. Genes were classified based on the following GO terms: “Extracellular space, GO:0005615,” “Extracellular region, GO:0005576” as localized at the extracellular, “Endoplasmic reticulum, GO:0005783” as localized at the endoplasmic reticulum. The classification was conducted for each phylogenetic class.

### Tissue-Specific Expression Analysis

The tissue-specific gene expression data were retrieved from the RefEx (Reference Expression dataset). This database was used to construct gene expression data for each human tissue based on the microarray and RNA-seq data. Moreover, tissue-specific gene expression data from 40 human tissues and organs were calculated using the ROKU algorithm, and tissue-specific patterns were assigned as follows: 1 for over-expressed outliers, −1 for under-expressed outliers, and 0 for non-outliers. The over-expressed information by RNA-seq was retrieved and visualized using a heatmap with the phylogenetic profile (18).

### Metabolic Pathway Analysis

Metabolic pathway data from the KEGG PATHWAY database were extracted using KEGG Mapper (19). The hierarchical clustering classified molecules were then mapped to metabolic pathways, followed by the evolutionary analysis of the metabolic pathways of proteins that interact with ORF8 and their interactors.

## Results

### Phylogenetic Profiling

**Figure 1:**
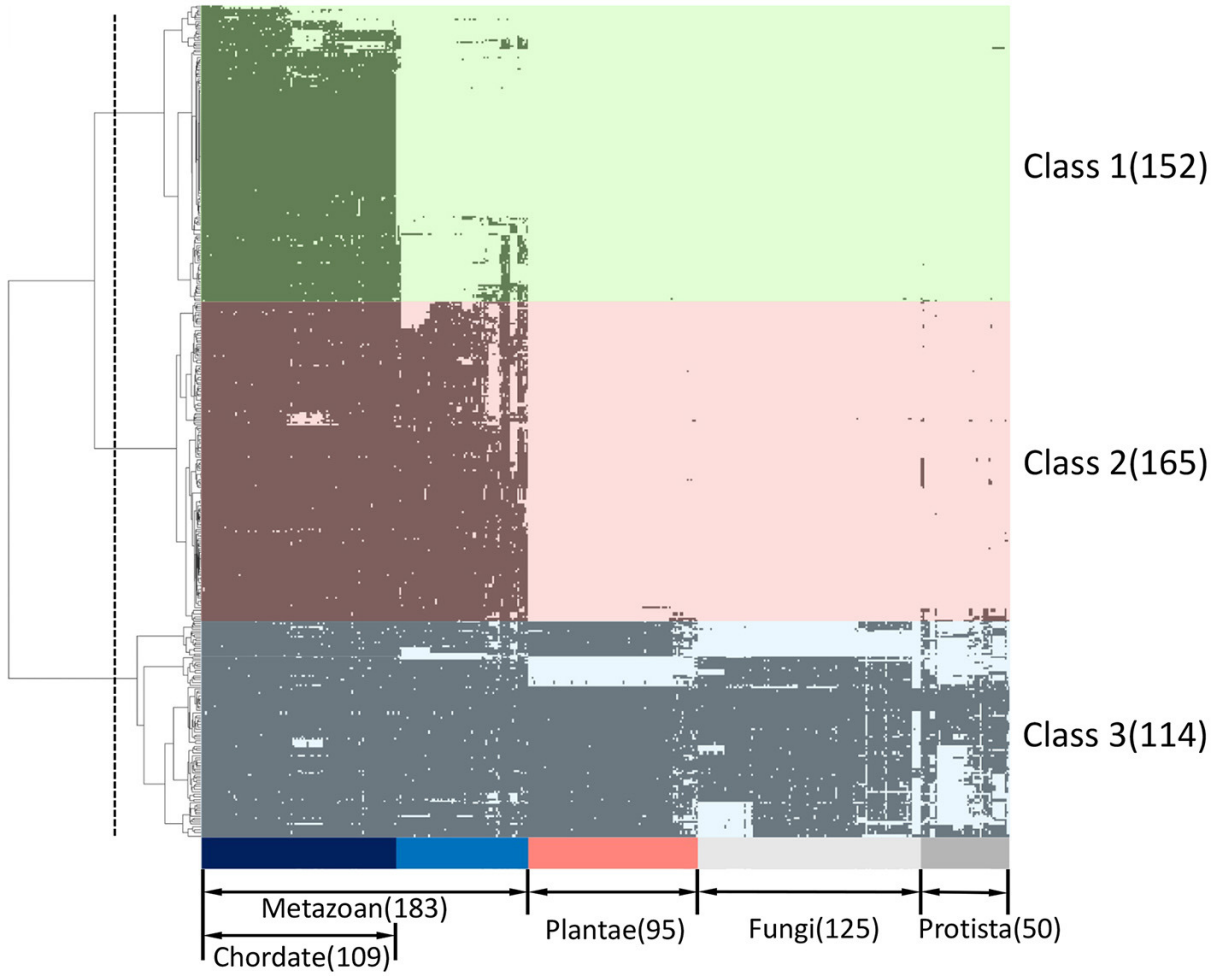
Phylogenetic profiling of ORF8-binding proteins and their interactors. The horizontal axis shows 453 eukaryotes for which whole genome sequences are available, and the vertical axis shows 431 human proteins related to ORF8. Proteins are indicated in black if orthologs are present in each species. The proteins were classified into three groups (Class 1–3) based on clustering analysis. The classes conserved across chordates (light green), metazoans (light pink), and eukaryotes (light blue) are shown. The dashed line indicates the threshold for hierarchical clustering. See Supplementary Table 1 for the detailed information.

We developed a binary matrix based on the presence (1) and absence (0) of ORF8-binding partners and their interactors in 453 eukaryotic species for phylogenetic profiling; cluster analyses were performed to determine the degree of co-evolution among the ORF8-interacting proteins and their interactors. The dataset was divided into three clusters, which were defined as Classes 1–3. A total of 152, 163, and 114 proteins were conserved in the chordates (Class 1), metazoans (Class 2), and eukaryotes (Class 3), respectively (Figure 1; Supplementary Table 1). If multiple homologous genes were involved in the different classes, we selected the most widely and evolutionarily conserved proteins to classify the proteins into a class.

### GO Analysis and Detection of Cellular Localization

**Figure 2:**
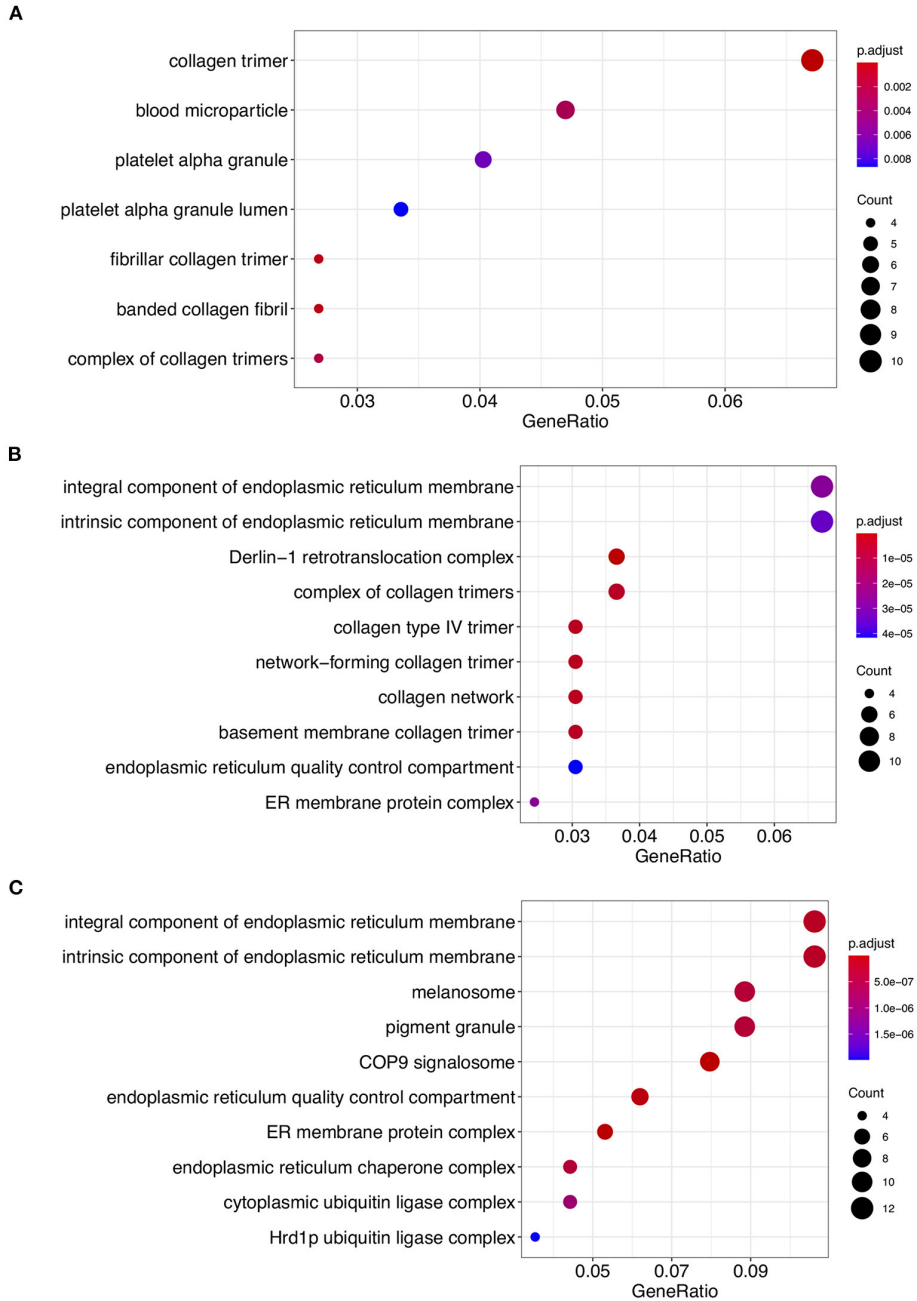
Gene ontology (GO) enrichment analysis of Class 1, Class 2, and Class 3 proteins. GO cellular component process terms that were significantly enriched in Class 1 **(A)**, Class 2 **(B)**, and Class 3 **(C)** are shown. The dots indicate enriched scores: red, high enrichment; blue, low enrichment. The sizes of the dots represent the number of genes in each row. The *x*-axis represents the percentage of genes involved in each row. See Supplementary Tables 2–4 for the detailed information.

We conducted GO enrichment analysis for each phylogenetic classification using phylogenetic profiling. The GO terms associated with cellular components related to the endoplasmic reticulum, including the “Integral component of endoplasmic reticulum membrane, GO:0030176” and “Intrinsic component of endoplasmic reticulum membrane, GO:0031227” were enriched in Class 2 and Class 3 (Figures 2B,C; Supplementary Tables 3, 4). On the other hand, the GO terms “collagen trimer, GO:0005581,” “Blood microparticle, GO:0072562,” and “Platelet alpha granule, GO:0031091,” were enriched in Class 1 (Figure 2A; Supplementary Table 2). These results imply that Class 2 and Class 3 proteins might be involved in endoplasmic function. In contrast, it appears that Class 1 proteins might be involved in collagen-mediated extracellular regulation and platelet granule regulation.

**Figure 3:**
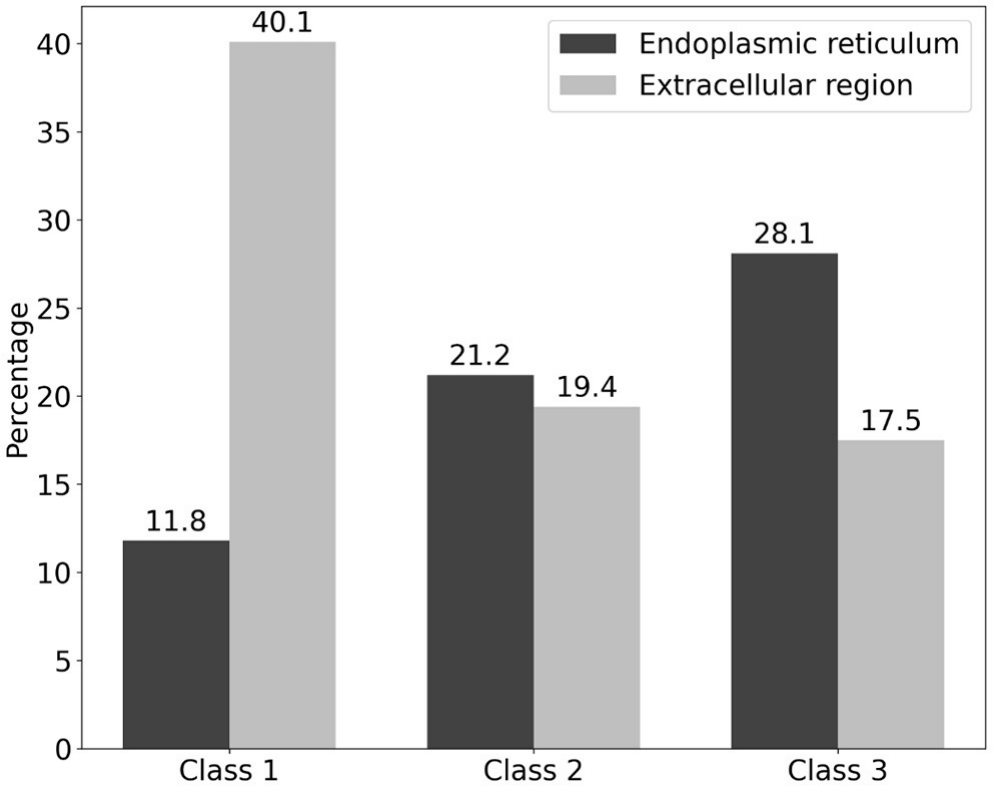
Classification of cellular localization of the three classes of the ORF8-binding proteins and their interactors. The localization of ORF8-binding proteins and their interactions is shown. The vertical axis represents the percentage of each class. The percentage of proteins localized in the endoplasmic reticulum (black) and extracellular region (silver) is shown. Proteins that had both localization terms were also included. See Supplementary Table 1 for the detailed information.

We classified the cellular anatomical region by focusing on the extracellular region and endoplasmic reticulum using the GO cellular component (Supplementary Table 1). With respect to Class 1, the percentage of proteins that were localized in the extracellular region was higher (40.1%) than that of the proteins in the other two classes (Figure 3). In contrast, the percentage of proteins localized in the endoplasmic reticulum was lower (11.8%) in Class 1 compared to the other classes (Figure 3). These results are consistent with the previously published results (9). In contrast, proteins from Classes 2 and 3 showed a tendency for ER localization compared to extracellular proteins, with a very slight difference for Class 2. Even though the localization tendency is not as apparent as in Class 1, the role of Classes 2 and 3 proteins in ER is supported by GO enrichment analysis. To further clarify the biological processes associated with the two specified localizations, we performed GO enrichment analysis on protein members of each localization in each class. GO enrichment analysis indicated that protein maturation and endopeptidase-related extracellular proteins can be categorized according to the GO terms of Class 1 endoplasmic reticulum proteins. Protein maturation, collagen fibril formation, and protein processing were enriched in the GO terms of extracellular region proteins. Comparison of the GO terms of extracellular region proteins between Class 2 and Class 3 indicated that although *p*-adjust values were slightly low, the term “cartilage development” were enriched only in Class 2 (Supplementary Figure 1). In contrast, GO terms “ERAD” and “protein folding” were commonly enriched in the endoplasmic reticulum of the Class 2 and the Class 3 proteins (Supplementary Figure 2).

### Tissue Specific Expression Analysis

**Figure 4:**
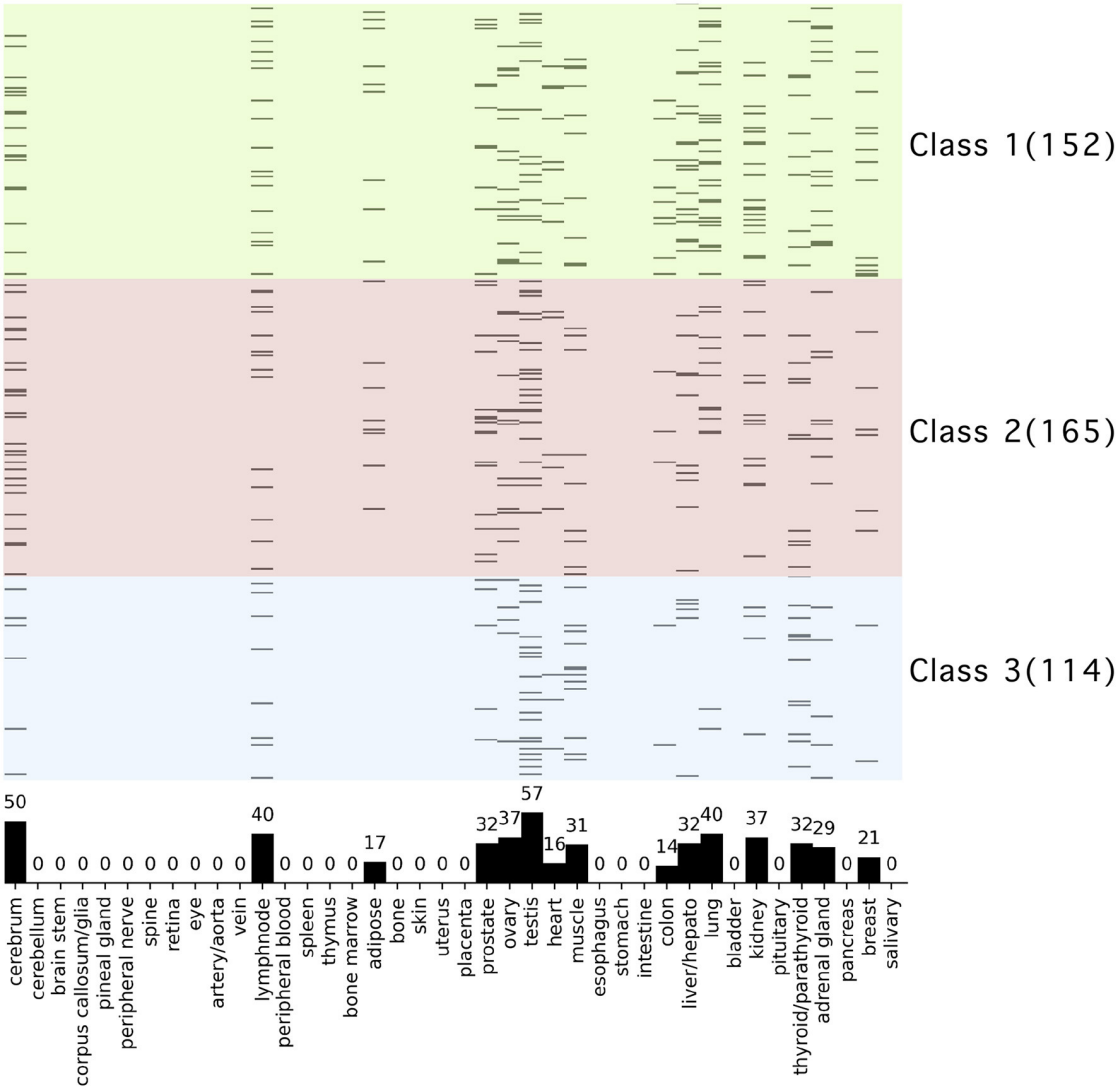
Tissue and organ specific expression analysis of ORF8-binding proteins and their interactors. The vertical axis shows 431 human proteins related to ORF8 proteins, and the background color indicates the phylogenetic cluster, as shown in Figure 1. The horizontal axis shows the 40 tissues and organs. Proteins are shown in black if tissue-specific gene expression is detected. The bar graph in the plot area shows the total number of genes with specific expression in each tissue. See Supplementary Table 5 for raw data.

We performed tissue-and organ-specific gene expression analysis of the proteins that interact with ORF8 and their interactors using data that had already been calculated based on the RefEx database. The tissue specific expression was observed in the following tissues: cerebrum, lymph node, adipose, prostate, ovary, testis, heart, muscle, colon, liver/hepato, lung, kidney, thyroid/parathyroid, adrenal gland, breast (Figure 4). The highest number of proteins in terms of tissue specific expression was found in testis (57). The second and third highest values were cerebrum (50), lung (40), and lymph node (40). We confirmed the evolutionary trends of the proteins localized in specific tissues. The tissue terms liver hepato, lung, and kidney were enriched in the genes involved in Class 1 (conserved in chordates). In contrast, these terms were not enriched in the proteins involved in Classes 2 and 3 (Supplementary Table 5). It should be noted that the profile of proteins in this study was extended from the STRING database based on ORF8-interacting proteins from HEK 293T cells alone. Hence, the tissue-specific expression analysis here might produce a bias toward specific tissues.

### KEGG Pathway Analysis

We performed KEGG pathway analysis against the proteins in each class and indicated these with different colors when mapping them to the specific biological pathways in order to analyze the functions of the ORF8-binding proteins and their interactors during evolutionary classification. The following pathways were found to be enriched: “Protein processing in endoplasmic reticulum” (50 proteins), “Human papillomavirus infection” (41 proteins), “Pathways in cancer” (27 proteins), “Focal adhesion” (23 proteins), “PI3K-Akt signaling pathway” (22 proteins), and “ECM-receptor interaction” (18 proteins).

**Figure 5:**
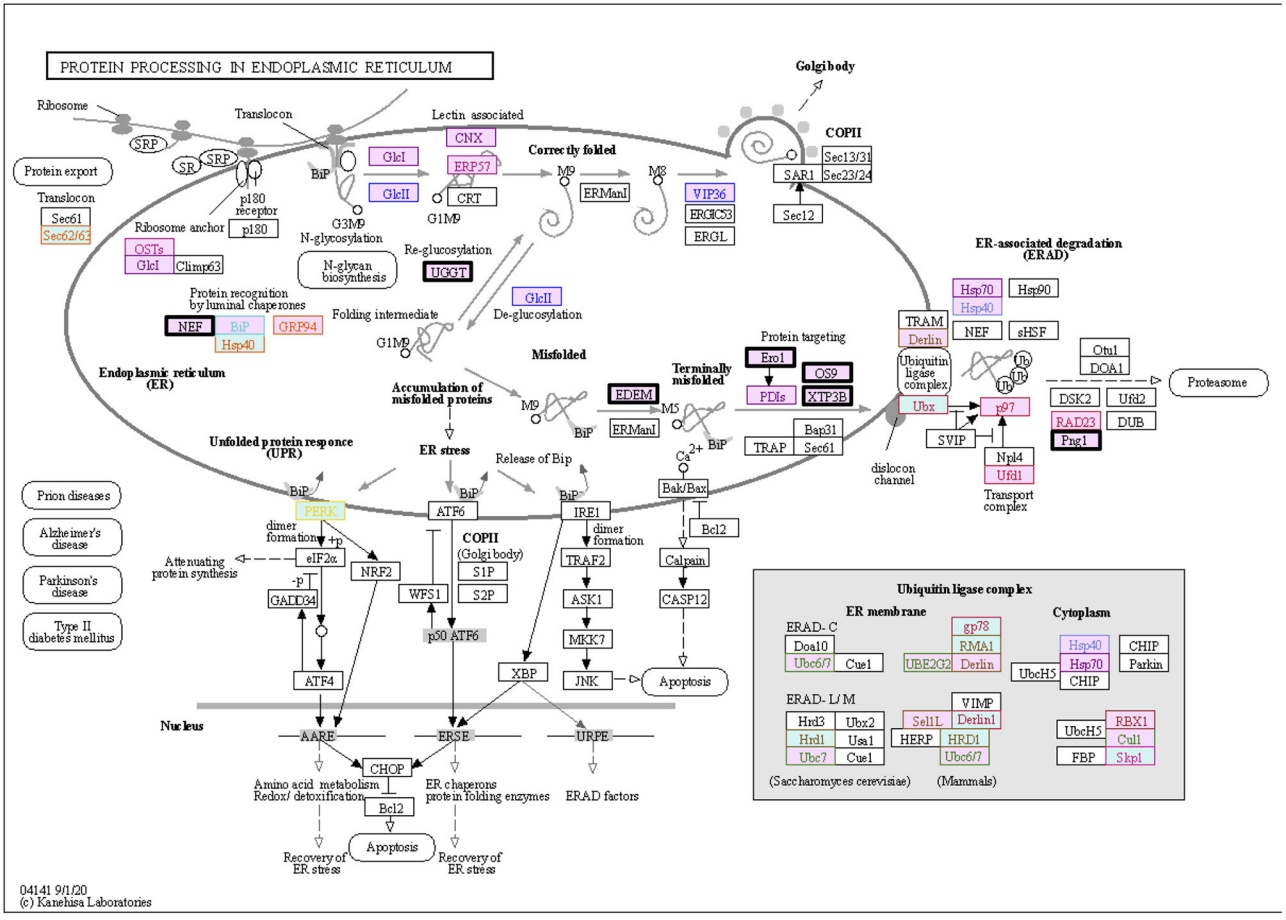
ORF8-binding proteins and their interactors expressed in the “Protein Processing in Endoplasmic Reticulum” pathway. The proteins in the dataset that interact with ORF8 and its binding proteins are shown in the KEGG PATHWAY map. Squares represent proteins or genes. The solid arrows represent direct interactions, and dashed lines represent indirect interactions. The background color indicates the class of phylogenetic clusters: yellow, Class 1; light blue, Class 2; light pink, Class 3. If multiple homologous genes were involved in a different class, we selected the most widely and evolutionarily conserved proteins to classify the proteins into a class. The proteins that interact directly with ORF8 are depicted in bold black frames. Other text colors, including orange, yellow, red, ocher, pink, dark red, purple, sky blue, blue, dark blue, green, or brown indicate interactions with HYOU1, PLD3, NGLY1, ERLEC1, EMC1, ERO1LB, UGGT2, SIL1, EDEM3, ERP44, FBXL12, and POFUT1, respectively. See Supplementary Table 6 for detailed information on mapped colors.

**Figure 6:**
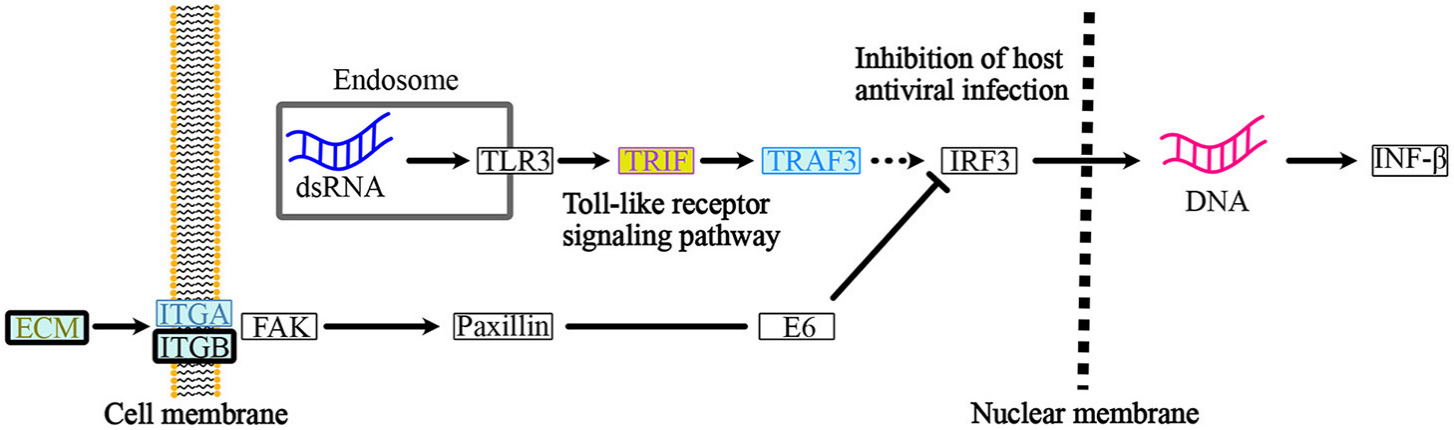
ORF8-binding proteins and their interactors in the “Human Papillomavirus Infection” pathway. Within the endosome of the host cells, dsRNA from the infected RNA virus, SARS-CoV-2 triggers the “toll-like receptor signaling pathway”. PVR-interacting protein: TRIF (Class 1) and IL17RA-interacting protein TRAF3 (Class 2) are involved in this pathway. The host cells respond and produce IFN-β to activate the “inhibition of antiviral infection”. Integrin signaling acts as a negative regulator of the “toll-like receptor signaling pathway” to regulate the IFN-β-mediate innate immune response. PLOD2, COL6A1, LOX, and PVR-interacting proteins: ECM (Class1 and Class 2), ITGB-1, and PVR-interacting proteins: ITGA (Class 2) and ITGB (Class 2) are involved in this integrin pathway. See Supplementary Figure 3 for a depiction of the entire pathway. The background color indicates the class of the phylogenetic cluster, as shown in Figure 5. The proteins that interact with PLOD2, PVR, ITGB1, or IL17RA are indicated with gold, light purple, smoke blue, or brilliant blue text color, respectively. See Supplementary Table 7 for detailed information on mapped colors.

**Figure 7:**
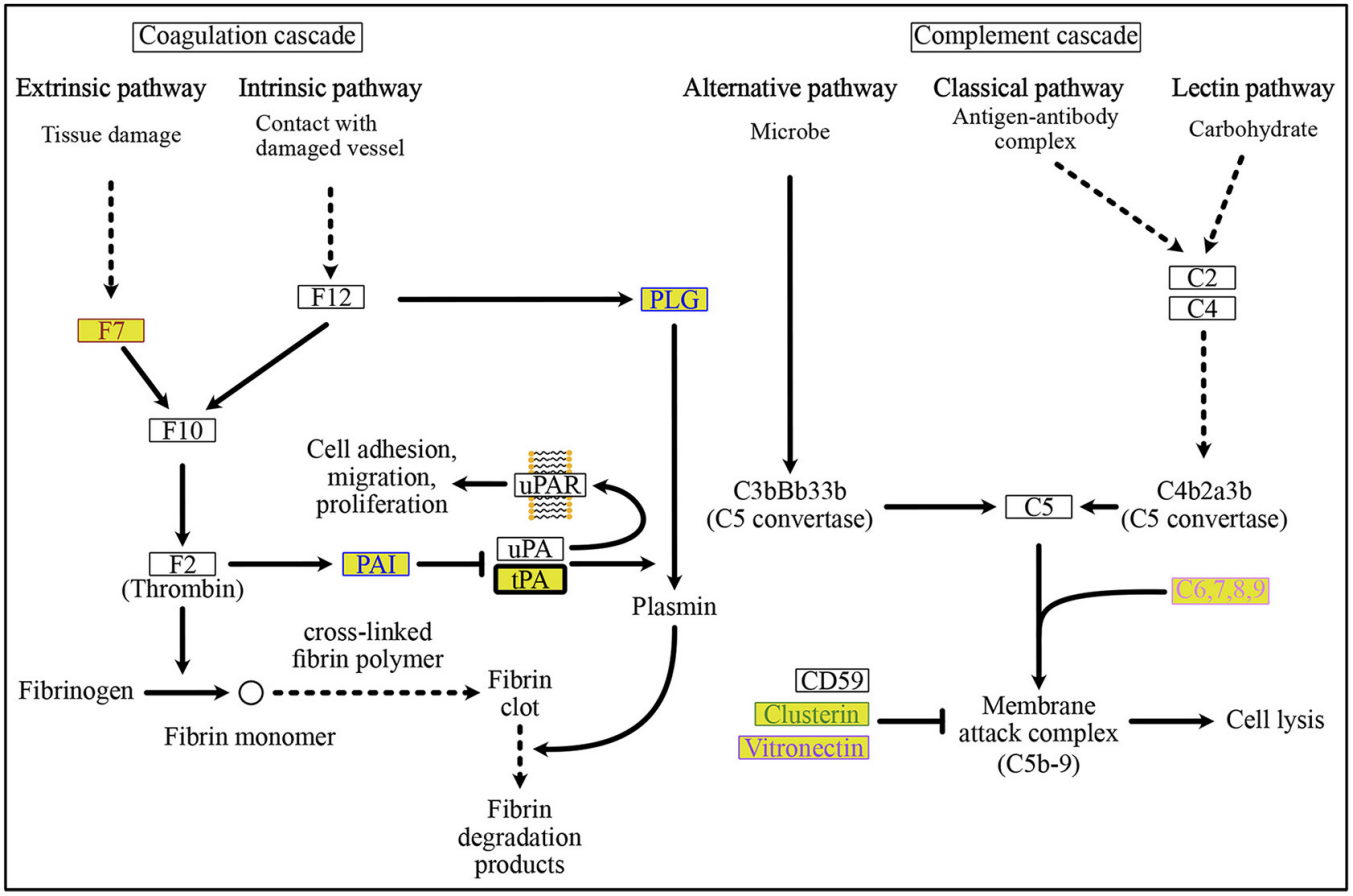
ORF8-binding proteins and their interactors in the “Complement and Coagulation Cascades” pathway. Plasmin-mediated fibrin degradation, called “fibrinolytic system” is the main process of coagulation. The PLAT-interacting protein, PAI and ORF8-binding protein, tPA, are involved in this “fibrinolytic system”. PAI is a negative regulator of tPA-dependent activation of the “fibrinolytic system” to remove fibrin clots. Membrane attack complex-mediated cell lysis occurs in the complement-mediated immune lysis cascade. See Supplementary Figure 4 for a depiction of the pathways. The background colors indicate the class of phylogenetic clusters, as shown in Figure 5. The text color indicates the interactors as follows: POFUT1 interactors are colored brown, PLAT interactors are colored blue, MFGE8 interactors are colored yellow green, PVR interactors are colored light purple, and CHPF interactors are colored by prism pink. C6,7,8,9 interacts with the ORF8-binding proteins CHPF and GDF15. See Supplementary Table 8 for detailed information on mapped colors.

The KEGG PATHWAY “Protein processing in endoplasmic reticulum” includes 7 ORF8-binding proteins conserved in Class 3 and 29 of their interactors (Figure 5). By focusing on their interaction factors, the proteins that form the ubiquitin ligase complex could be identified. Most of these proteins are conserved in animals (Class 2 and Class 3). Focusing on the second most mapped pathway, human papillomavirus infection (Figure 6; Supplementary Figure 3), the identified proteins included those that are associated with signaling from toll-like receptor 3 (TLR3) that recognize the dsRNA received from endosomes to interferon beta (INF-β) that suppress viral infection. In this pathway, TIR-domain-containing adapter-inducing interferon-β (TRIF) is conserved in Class 1 and interacts with the poliovirus receptor (PVR), which binds directly to ORF8, and TRIF downstream of it is conserved in Class 2 and interacts with interleukin 17 receptor A (IL17RA), which binds directly to ORF8. This pathway is negatively regulated by ECM (Class 1 and Class 2) and integrin (Class 2)-mediated integrin signaling. There are two independent pathways in the complement and coagulation cascades (Figure 7; Supplementary Figure 4). One is blood clotting through the plasmin via tissue-type plasminogen activator (PLAT), which binds directly to ORF8, plasminogen, and plasminogen activator inhibitor. The other is the complement, which activates cell lysis and vitronectin, which inhibits it. CHPF- and GDF15-interacting proteins, C6, C7, C8, and C9 (all of which are involved in Class 1)-mediated promotion of membrane attack complex formation triggering the cell lysis process. In contrast, both MFGE8-interacting protein, clusterin (Class 1) and PVR-interacting protein, vitronectin (Class 1), are negative regulators of the membrane attack complex formation.

## Discussion

Next-generation sequencing has resulted in the proliferation of genomic data from diverse species. This enormous quantity of genomic data has made possible, through comparative genomics, a number of advances in the elucidation of gene function and crosstalk. One such approach is phylogenetic profiling, an independent technique to infer functionally related genes and protein-protein interactions via the correlation of the occurrence across a set of genomes or so-called profiles that show the degree of co-evolution between genes (9). Phylogenetic profiling relies on the hypothesis that genes that function together are associated with similar evolutionary pressures and are thus lost and gained together throughout evolution (20). Genes with close relationships in the cluster tree developed from phylogenetic profiling are considered to share common functionality and pathways.

The ORF8 accessory protein is a SARS-CoV-2 viral protein that displays numerous fascinating features. The genomic region that encodes this protein has been recognized as one of the most variable regions of SARS-CoV-2, as well as a recombination hotspot and a region highly susceptible to deletions and nucleotide substitutions (2, 21). Consequently, among all SARS-CoV-2 viral proteins, ORF8 shares the least homology with other known coronaviruses. Previously, we performed phylogenetic profiling and clustering analyses of proteins that are physically associated with SARS-CoV-2 ORF8, as determined by affinity purification mass spectrometry assays (7). We reported that ORF8-interacting proteins can be classified into three classes, which are conserved in vertebrates (Class 1), metazoans (Class 2), and eukaryotes (Class 3) based on their history of evolutionary pressure. We later identified the associated function of each cluster and suggested that Class 1 might contribute to viral pathogenesis and Classes 2 and 3 might contribute to the immune evasion mechanism. Generally, we suggest that ORF8 is associated with SARS-CoV-2 immune evasion and pathogenicity (9). Experimentally, ORF8 was reported to bypass the host immune surveillance mechanism by downregulating HLA class I and inhibiting IFN-β production (8, 10). This viral protein has also been reported to induce the ER stress pathway (10). Additionally, the deleted ORF8 variant of SARS-CoV-2 is known to have milder clinical illness (6).

In the present study, we expanded our previous work on ORF8 phylogenetic profiling to identify the interactors of ORF8-interacting proteins. We found that these proteins are evolutionarily clustered into three classes that are conserved across chordates (Class 1: 152 proteins), metazoans (Class 2: 163 proteins), and eukaryotes (Class 3: 114 proteins). Subsequently, we identified the localization profile, tissue-specific expression, and KEGG pathway of the protein members in each class. Following these analyses, we found that Class 1 was enriched in extracellular proteins and proteins with tissue-specific expression in the liver, lungs, and kidneys. KEGG pathway analysis showed that proteins from Class 1 were strongly associated with the complement and coagulation cascade pathways. This mechanism covers two independent pathways, including the complement, which is part of the innate immune system and coagulation, which is essential for preventing excessive bleeding through clot formation. ORF8 binds to PLAT, a plasminogen activator that induces blood clotting, and is indirectly associated with vitronectin, a cytolytic complement inhibitor, through PVR. Although the mechanism is unclear, we postulate that ORF8 partly contributes to COVID-19 pathogenesis via this system. Moreover, some proteins in Class 1, including IL17RA, growth differentiation factor 15, FK506-binding protein 10, and PLAT, are associated with sarcoidosis pathogenesis (22). Indeed, it is known that ORF8 plays a role in COVID-19 pathogenesis, and a variant with its absence leads to milder illness (6). It would be interesting to further evaluate the contribution of protein members in Class 1 to COVID-19 pathogenesis.

GO enrichment analyses indicated that Class 1 endoplasmic reticulum proteins and extracellular proteins act as protein processing regulators in ER and protein maturation collagen fibril formation in the extracellular region, respectively. Although the percentage of the GO terms, extracellular region, are similar between Class 2 and Class 3 proteins, we found the GO term “cartilage development” was concentrated only in the Class 2 proteins. This result implies that among the ORF8-interracting proteins, cartilage-related proteins acquired during evolution in the metazoans are correlated with ORF8-mediated COVID-19 in the cartilage. Further studies are required to investigate this possibility. In the endoplasmic reticulum, both Class 2 and Class 3 proteins are correlated with the ERAD pathway via interaction with the unfolded proteins.

Some proteins from Class 1, along with some proteins from Class 2, are also associated with the human papillomavirus infection pathway, especially TLR3 induced-signaling that yields the transcription and secretion of IFN-β for the host immune response. TLR3 is an important receptor that recognizes the dsRNA of viruses received from endosomes during viral infection. Here, ORF8 interacts with PVR, which is later associated with TRIF, downstream of TLR3. The consequences of ORF8 interaction are unknown. However, it has been reported that ORF8 can decrease the nuclear translocation of interferon regulatory factor 3 (IRF3), which is downstream of the TRIF pathway, thereby suppressing the interferon-stimulated gene (ISG) ISG15 and ISG56 and antagonizing IFN-β production (10). Additionally, PVR can also be associated with the integrin signaling pathway, which can act as a negative regulator of the TLR3 signaling pathway (Figure 6). It is conceivable that the inhibition of IFN-β is induced by ORF8 interaction via PVR. This hypothesis requires further evaluation.

The protein members of Class 3 in our phylogenetic profiling were mostly localized in the ER and were associated with protein processing in the ER based on the KEGG pathway. Viral infections commonly exploit the ER for replication, assembly, morphogenesis, and egression (23). ORF8 has been reported to induce the ER stress pathway and the unfolded protein response (UPR) by activating the ATF6 and IRE1 pathways (10). This mechanism promotes persistent infection by maintaining the survivability of infected cells during apoptosis. However, UPR also regulates immune cell differentiation, activation, and cytokine production. It is unclear whether ORF8 can exploit the UPR system to maintain SARS-CoV-2 survivability and evade the resulting immune surveillance. Additionally, ORF8 can also mediate HLA 1 degradation by hijacking the Beclin 1 autophagy initiation pathway, thereby protecting the host cell against cytotoxic T lymphocytes (8). Beclin 1 is a member of Class 3 in our phylogenetic profiling. Taken together, the findings of the present study, along with previous research, elucidate the immune evasion mechanism of SARS-CoV-2 through ORF8 in the ER.

Recently, in a study of mutational changes in SARS-CoV-2, it was reported that a fraction of the Omicron variant acquired a mutation in the ORF8 encoding region (24). Because ORF8 is associated with immune evasion and pathogenicity, further studies to investigate the characteristics of this protein within the omicron variant are important.

## Conclusion

Through phylogenetic profiling, we have classified ORF8 associated proteins based on their evolutionary history and pressures and, further, we explored the trend of mechanisms that are exploited or associated with ORF8 in evading the immune system as well as contributing to COVID-19 pathogenesis. We predict that the protein members in Class 1 could contribute to COVID-19 pathogenesis via complement and coagulation cascades and promote sarcoidosis. Additionally, proteins in Classes 1 and 2 may contribute to the downregulation of IFN-β. Finally, we postulate that the proteins in Class 3 are associated with ER stress and mediate HLA 1 degradation. Notably, the analyses performed in this study are based entirely on a bioinformatics approach; thus, the conclusions presented here are purely predictions that requires further validation.

## Data Availability Statement

The datasets presented in this study can be found in online repositories. The names of the repository/repositories and accession number(s) can be found in the article/Supplementary Material.

## Author Contributions

HT, MF, YK, and MI conceived the project and wrote the manuscript. HT, KH, and TS performed the experiments and acquired and analyzed the data. HT, MF, KH, and MI performed the data analysis. All authors contributed to the article and approved the submitted version.

## Funding

This study was funded by the Takeda Science Foundation.

## Conflict of Interest

The authors declare that the research was conducted in the absence of any commercial or financial relationships that could be construed as a potential conflict of interest.

## Publisher's Note

All claims expressed in this article are solely those of the authors and do not necessarily represent those of their affiliated organizations, or those of the publisher, the editors and the reviewers. Any product that may be evaluated in this article, or claim that may be made by its manufacturer, is not guaranteed or endorsed by the publisher.
