# Supplementary material for: In silico Analysis of SARS-CoV-2 ORF8-Binding Proteins Reveals the Involvement of ORF8 in Acquired-Immune and Innate-Immune Systems
Source: Front Med (Lausanne). 2022 Feb 1;9:824622. doi: 10.3389/fmed.2022.824622 (PMC8844466; doi:10.3389/fmed.2022.824622)
Supplement: Supplementary file 1 [file Data_Sheet_1.zip › Supplementary_Material/Supplementary_Material.docx]

Supplementary Material

**Supplementary Figure 1. GO enrichment analysis of Class 1, Class 2 and Class 3 extracellular region proteins.**

GO biological process terms that were significantly enriched in the top ten are shown. The dots indicate enriched scores: red indicates high enrichment, and blue indicates low enrichment. The sizes of the dots represent the number of genes in each row. The x-axis represents the percentage of genes involved in each row. GO terms of the Class 1 (A), Class 2 (B) and Class 3 (C) extracellular region proteins.

**Supplementary Figure 2. GO enrichment analysis of Class 1, Class 2 and Class 3 endoplasmic reticulum proteins.**

GO biological process terms that were significantly enriched in the top six or ten are shown. The dots indicate enriched scores: red indicates high enrichment, and blue indicates low enrichment. The sizes of the dots represent the number of genes in each row. The x-axis represents the percentage of genes involved in each row. GO terms of the Class 1 (A), Class 2 (B) and Class 3 (C) endoplasmic reticulum proteins.

**Supplementary Figure 3. ORF8-binding proteins and their interactors expressed in the “Human Papillomavirus Infection” pathway**

The proteins in the dataset were mapped identically as Figure 4. The text color indicates the profile of interactors as follows: the of interactors of NPC2, NGLY1, DNMT1, PLOD2, PVR, FKBP10, ITGB1, IL17RA, and PLEKHF2 are colored by vivid pink, red, turquoise blue, gold, light purple, reddish brown, smoke blue, brilliant blue, and forest green, respectively. ECM interacts with ORF8-binding proteins, including PLOD2, COL6A1, PVR and LOX, while ITGA interacts with ORF-binding proteins, including ITGB1 and PVR. See Supplementary Table 7 to find out the detailed information of the mapped colors.

**Supplementary Figure 4. ORF8-binding proteins and their interactors expressed in the “Complement and Coagulation Cascades” pathway**

The proteins in the dataset were mapped identically as Figure 4. The text color indicates the profile of interactors as follows: the of interactors of POFUT1, PLAT, MFGE8, PVR, and CHPF are colored by brown, blue, yellow green, light purple, and prism pink, respectively. C6,7,8,9 interacts with ORF8-binding proteins, including CHPF and GDF15. See Supplementary Table 8 to find out the detailed information of the mapped colors.

**Supplementary Table 1. The phylogenetic classification and additional information of the ORF8-binding proteins and their interactors**

The table includes the information of set of proteins used in this study and the classes clustered by phylogenetic profile, classification of cellular component, and annotation information obtained from UniProt.

**Supplementary Table 2. GO enrichment terms of the phylogenetic profiling to the Class 1 in ORF8-binding proteins and their interactors**

The table includes GO terms (BP: Biological Process, CC: Cellular Component, MF: Molecular Function) that are enriched in Class 1.

**Supplementary Table 3. GO enrichment terms of the phylogenetic profiling to the Class 2 in ORF8-binding proteins and their interactors**

The table includes GO terms (BP: Biological Process, CC: Cellular Component, MF: Molecular Function) that are enriched in Class 2.

**Supplementary Table 4. GO enrichment terms of the phylogenetic profiling to the Class 3 in ORF8-binding proteins and their interactors**

The table includes GO terms (BP: Biological Process, CC: Cellular Component, MF: Molecular Function) that are enriched in Class 3.

**Supplementary Table 5. Tissue specific gene expression of the ORF8-binding proteins and their interactors**

Forty tissue-specific genes are indicated by “1” and non-tissue-specific genes are indicated by “0”.

**Supplementary Table 6. Color annotation in the “Protein Processing in Endoplasmic Reticulum” pathway**

The table describes the color information of the Figure 5.

**Supplementary Table 7. Color annotation in the “Human Papillomavirus Infection” pathway**

The table describes the color information of the Figure 6 and Supplementary Figure 3.

**Supplementary Table 8. Color annotation in the “Complement and Coagulation Cascades” pathway**

The table describes the color information of the Figure 7and Supplementary Figure 4.
