# Supplementary figures and images for: In silico Analysis of SARS-CoV-2 ORF8-Binding Proteins Reveals the Involvement of ORF8 in Acquired-Immune and Innate-Immune Systems
Source: Front Med (Lausanne). 2022 Feb 1;9:824622. doi: 10.3389/fmed.2022.824622 (PMC8844466; doi:10.3389/fmed.2022.824622)

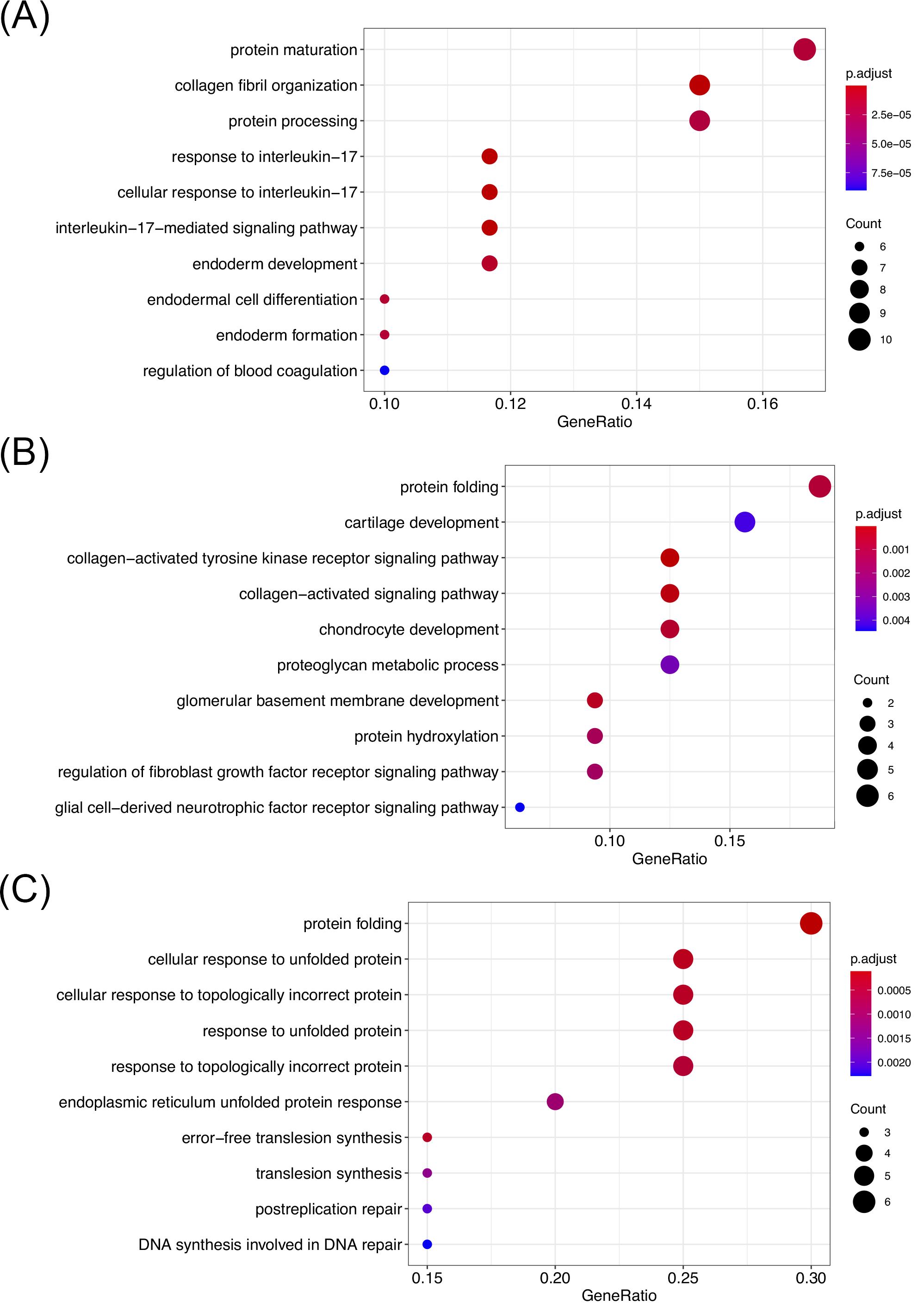

Supplement: Supplementary file 1 [file Data_Sheet_1.zip › Supplementary_Material/Supplementary Figure 1.tif]

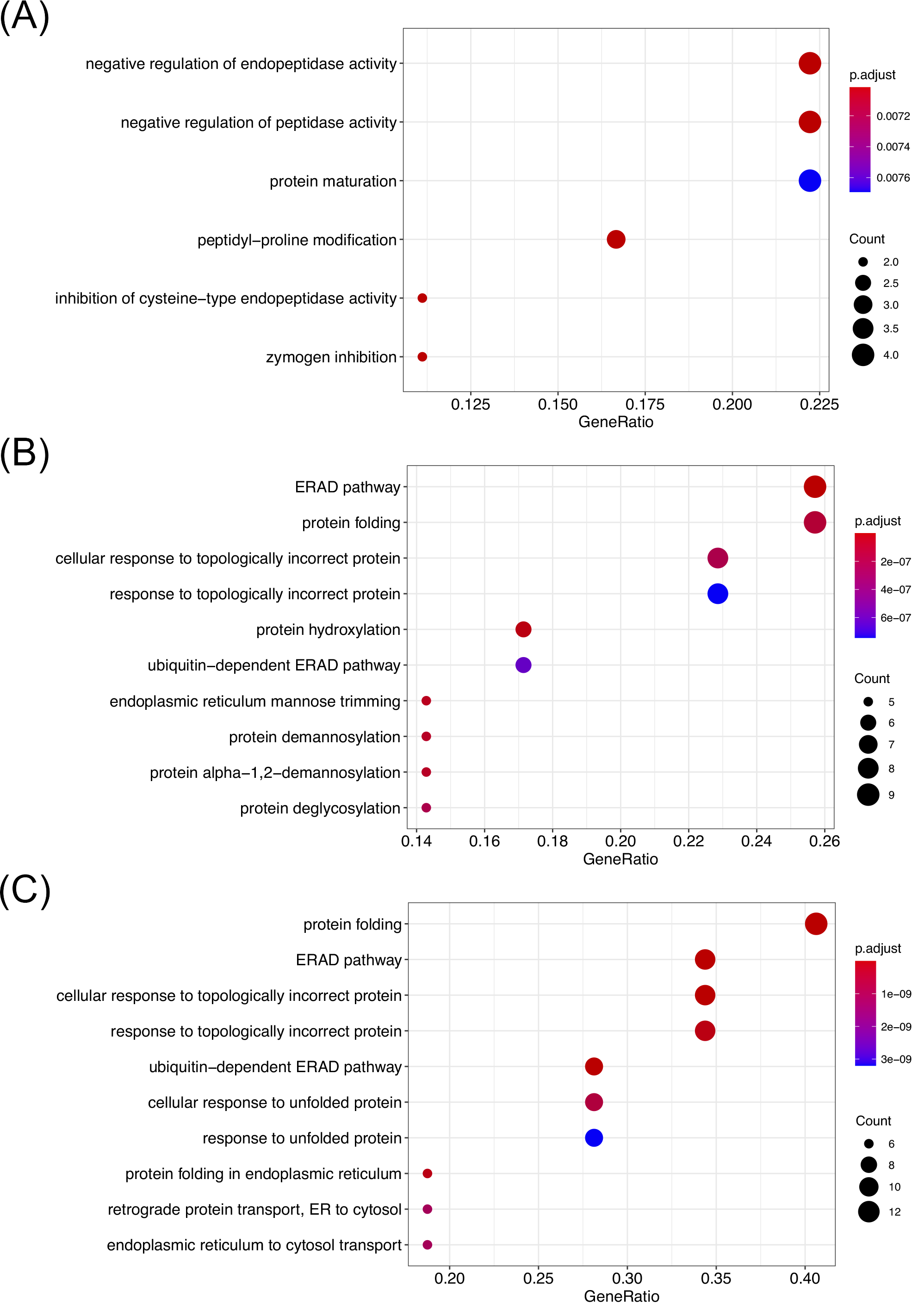

Supplement: Supplementary file 1 [file Data_Sheet_1.zip › Supplementary_Material/Supplementary Figure 2.tif]

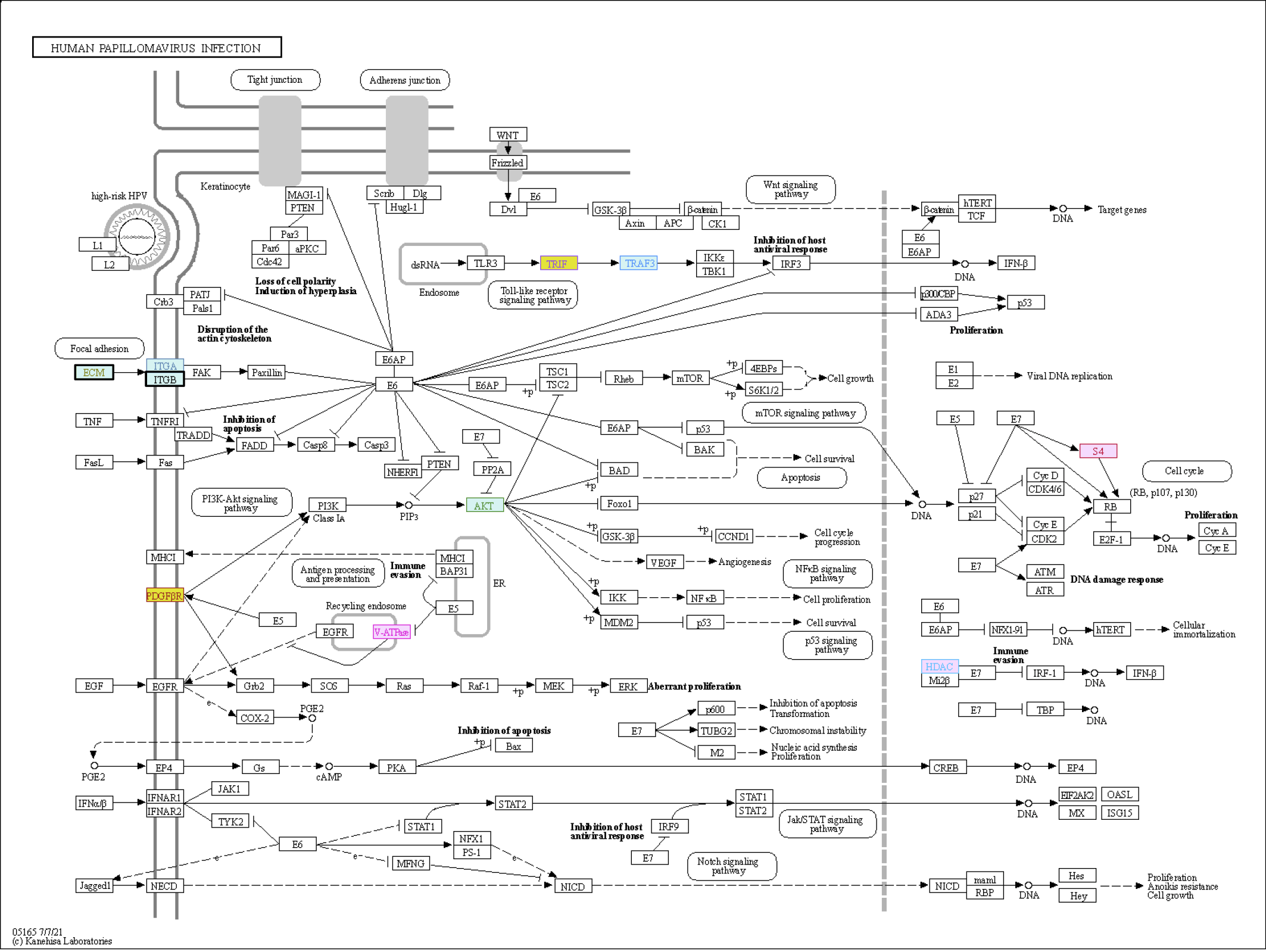

Supplement: Supplementary file 1 [file Data_Sheet_1.zip › Supplementary_Material/Supplementary Figure 3.tif]

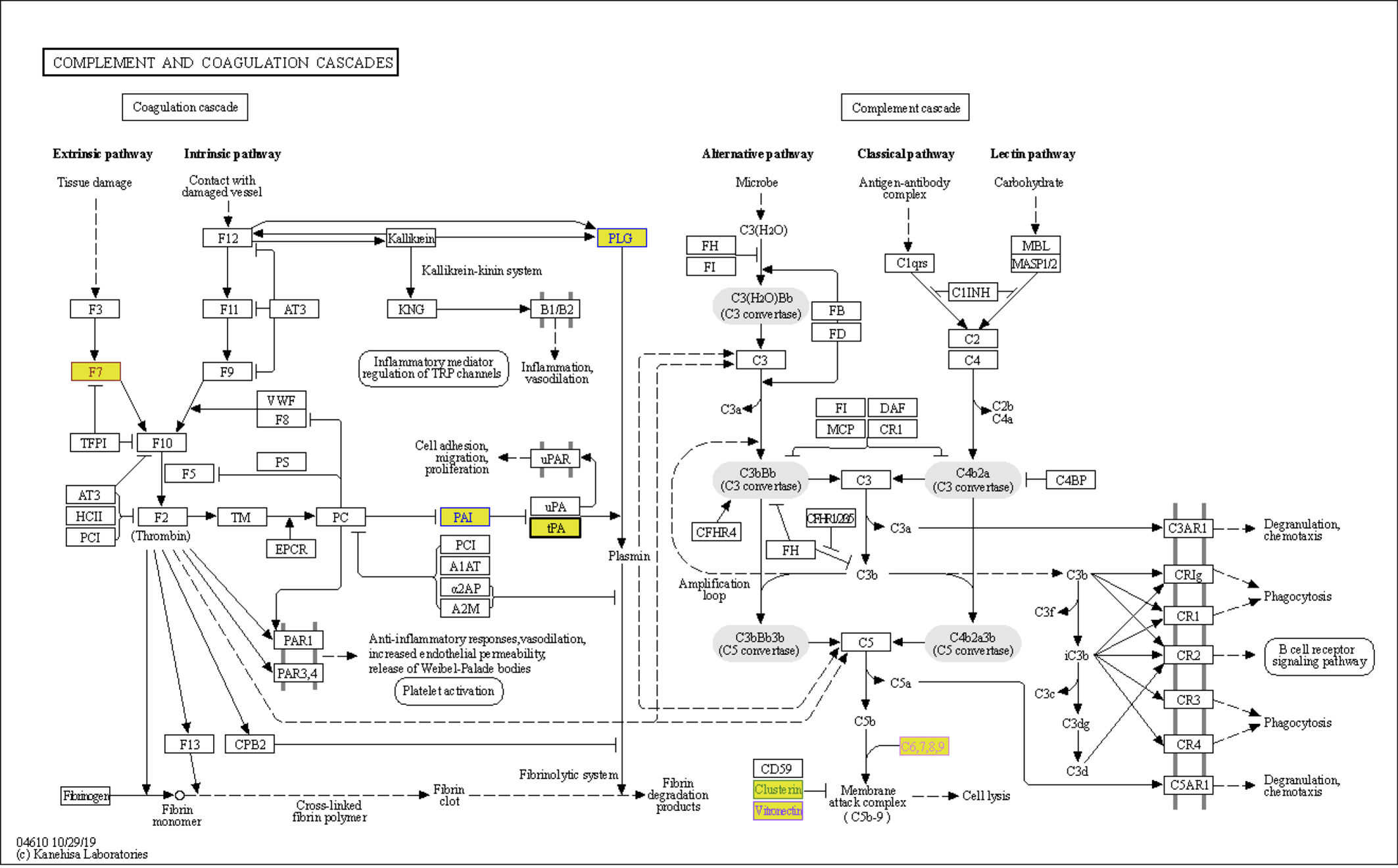

Supplement: Supplementary file 1 [file Data_Sheet_1.zip › Supplementary_Material/Supplementary Figure 4.tif]
